# State of viral hepatitis knowledge and testing uptake in Brazil: Findings from the National Survey of Knowledge, Attitudes and Practices (PCAP-2013)

**DOI:** 10.1186/s41124-016-0003-y

**Published:** 2016-04-14

**Authors:** Silvano Barbosa de Oliveira, Meritxell Sabidó, Ana Roberta Pati Pascom, Juliana Machado Gisiviez, Adele Schwartz Benzaken, Fábio Mesquita

**Affiliations:** 1 Department of STI, AIDS, and Viral Hepatitis, Brazil Ministry of Health, Brasília, DF Brazil; 2 Fundação de Medicina Tropical Doutor Heitor Vieira Dourado (FMT-HVD), Avenida Pedro Teixeira 25, CEP: 69040-000 Manaus, AM Brazil

**Keywords:** Hepatitis, Viral, Knowledge, Testing, Brazil

## Abstract

**Background:**

Data were obtained from the third National Survey of Knowledge, Attitudes and Practices of HIV and other Sexually Transmitted Infections (STI) (PCAP-2013) and used to describe the current state of viral hepatitis (VH) knowledge and screening as well as the prevalence of viral hepatitis B (HBV) vaccination in Brazil and to assess the factors associated with testing uptake.

**Methods:**

A probability sample survey of 12,000 individuals (50 % men) aged between 15 and 64 years was conducted between October and December of 2013. The participants completed the survey in their own homes through computer-assisted face-to-face interviews and self-interviews. We analysed data related to self-reported knowledge of VH routes and screening uptake after weighting the variables to account for unequal selection probabilities and correct for differences in sex, age and region according to 2010 census figures.

**Results:**

The levels of correct knowledge regarding HBV and HCV transmission through unprotected sex were 33.1 and 34.3 %, respectively. The levels of correct knowledge regarding HBV and HCV transmission through tattooing/piercing were 26.4 and 24.5 %, respectively. Overall, 29 % of the respondents reported having underwent VH screening and 73.9 % reported prior HBV vaccination. VH screening was associated with the following factors: age between 25 and 49 years (adjusted male odds ratio (OR): 2.29, [95 % confidence interval (CI): 1.83–2.87]; female OR: 1.36, [95 % CI: 1.10-1.67]); age between 50 and 64 years (male OR: 1.52, [CI: 1.13–2.03]; female OR: 1.29, [CI: 1.02–1.63]); initial sexual intercourse before the age of 15 years in men (OR: 1.32, [CI: 1.10–1.57]); higher socioeconomic statuses of group A (male OR: 2.38, [CI: 1.81–3.13]; female OR: 2.10, [CI: 1.66–2.68]) and B (female OR: 1.56, [CI: 1.27–1.93]); and having ever been tested for HIV (male OR: 7.50, [CI: 5.82–8.53]; female OR: 7.13, [CI: 5.97–8.54]).

**Conclusions:**

This study revealed low levels of knowledge regarding VH transmission and screening practices in the general Brazilian population, especially among younger individuals and those with lower socioeconomic status. Efforts to enhance awareness campaigns and expand testing sites are needed to remove barriers to testing.

## Background

Globally, viral hepatitis (VH) affects approximately 500 million people and is responsible for 1.4 million deaths every year [1]. An estimated 57 % of cirrhosis and 78 % of hepatocellular carcinoma cases can be attributed to chronic hepatitis B or C infections [1]. In Brazil, a population-based survey conducted from 2005 to 2009 [2] revealed that 39.5 % of individuals aged between 5 and 19 years had been exposed to hepatitis A virus (HAV). The prevalence of individuals exposed to hepatitis B virus (HBV) (based on serological evidence of antibodies to hepatitis B core antigen [anti-HBc]) was 7.4 %, and the prevalence of those exposed to hepatitis C virus (HCV) was 1.4 %, although variation across regions was observed [3, 4]. The burden of chronic HBV has been estimated at 0.65 %, with 1 in 275,813 individuals being positive for hepatitis B surface antigen (HBsAg) [5]. Studies have also shown that the prevalence of exposure to hepatitis E virus (HEV) in adults is approximately 3 % [6], although little is known regarding the presence of HEV in the Brazilian population. Hepatitis D virus (HDV) infections in HBV cases have been observed in Amazonas [7]. In Brazil, the number of newly reported cases per 100,000 people in 2010 was 3.6 for HAV, 6.9 for HBV and 5.4 for HCV [8], and the annual number of deaths per 100,000 people was estimated at 0.03 for HAV, 0.3 for HBV and 1 for HCV [8]. Overall, 10 % of all deaths in Latin America (2008–2010) have been attributed to causes related to VH [9]. In Brazil, cirrhosis of the liver was ranked as the tenth leading cause of death in 2012 [10]. In Brazil, HCV infection is a major cause of cirrhosis, hepatocellular carcinoma (8040 new cases per year), and liver transplantation (30 % of transplants in 2011) [11].

In Brazil, VH response is integrated within the national HIV programme. The country has taken a leading role in raising awareness of VH at the intergovernmental level [12]. Policy regarding VH, as well as procurement of and payment for vaccination, falls under the responsibility of the Ministry of Health, and vaccination administration is free in Brazil’s municipalities. Domestic production of a HAV vaccine by public institutions has been planned [9]. Brazil has also introduced new efforts to expand the diagnosis and treatment of VH, and the country incorporated the use of rapid tests to improve early detection in 2011 [8]. Universal access to hepatitis treatment has been adopted, and clinical guidelines for HBV and HCV have been updated [13]. Several public media hepatitis campaigns have been undertaken, and since 2010 Brazil has commemorated World Hepatitis Day [13]. Despite these efforts, further improvements are still required. Knowledge and awareness of VH among the general population are limited, which might affect the adoption of preventative actions, such as testing uptake [14]. Indeed, most individuals with chronic hepatitis B or C infections are unaware of their status and remain untreated.

Over the past two decades, detailed information regarding the Brazilian population’s awareness of sexual health, HIV status and knowledge of other sexually transmitted infections (STIs) has been obtained through the National Survey of Knowledge, Attitudes and Practices (PCAP) [15, 16]. However, this large probability sample survey only included questions on VH in its third PCAP round (PCAP-2013). In the present study, we assessed the state of knowledge related to VH and prior testing for HBV and HCV and the factors associated with testing uptake in a general population sample from Brazil to provide actionable information for clinicians and policy makers.

## Methods

The PCAP-2013 survey interviewed a representative sample of 12,000 Brazilian men and women aged between 15 and 64 years between October and December of 2013. Brazil, one of the five largest countries in the world, has over 200 million inhabitants and a land area of more than 8,500,000 km^2^ [17].

### Sample design

The PCAP is a multistage, clustered and stratified probability sample survey. Census tracts with more than 60 permanent households were selected as primary sampling units (PSUs). Special sectors, such as military bases, boats, prisons, hospitals and convents, were excluded. Addresses within the census tracts were selected in the second stage, and only one eligible person per household was selected in the final stage. Prior to selection, the PSUs were stratified by region, main city, population density and rural versus urban contexts based on data from the 2010 census. The census tracts were selected systematically, and each sector was given a probability of selection that was proportional to its total number of households, with heads of households being stratified according to literacy level and education level of the sector being used as a proxy. Within each census tract, 16 households were systematically selected, and within each household, one person was selected based on sex, age group and cohabitation status [18].

The sample size of the PCAP study conducted in 2013 was calculated to provide robust estimates of the major parameters (e.g., consistent condom use with any type of partner, which was approximately 20 %) and a bilateral error of 2.5 %. Accounting for the complex sample design, the target sample size was set to 12,000 individuals. The sample was distributed by region, such that 1600 participants were from the north and central west, 2400 were from the south, and 3200 were from the northeast and southeast. Additionally, the sample contained equal numbers of men and women to provide sufficient statistical power to estimate the proportion of intravenous drug users, which was approximately 0.5 % with a bilateral error of 0.18 %.

### Data collection

Professional interviewers visited each address, established whether any of the residents were eligible, and selected one individual per household. Computer-assisted personal interviews were performed in the households. The more sensitive questions were previously validated through focus group discussions, and these were self-completed. The participants were asked questions related to the following factors: 1) sociodemographic status, including sex, age, education level and household assets; 2) sexual behaviours, including age at initial sexual intercourse, sexual encounters with members of the same sex, number of casual partners during the last year, and condom use during recent sexual intercourse; 3) drug use, including intravenous drug use; 4) VH transmission route knowledge; and, 5) self-reported hepatitis screening, including VH type, self-reported screening during the last 12 months, screening with the rapid test, screening sites, reasons for screening, knowledge of the test results, and self-reported vaccination history against HBV. Data were stored in a secured, password-protected location and they were encrypted before being sent online to the coordination centre.

### Study measures

To measure each participant’s socioeconomic status, a combination of educational level (completed/did not complete high school) and number of household assets (e.g., television, video player/recorder, radio, refrigerator, freezer, washing machine, dish-washing machine, fixed telephone, cellular phone, automobile, etc.) was used. Three socioeconomic status categories were established according to the Brazilian Association of Research Companies (ABEP) [19]. Group A/B individuals had six or more household assets and completed high school, group D/E individuals had fewer than six household assets and did not complete high school, and group C individuals were all remaining participants.

As knowledge indicators, we considered the percentages of individuals who correctly reported whether HBV or HCV can be transmitted via unprotected sexual intercourse (only HBV), needles, manicure/pedicure equipment, dental treatments, dialysis, endoscopy and tattooing or piercing. As a denominator, we used those who spontaneously reported that each route could transmit VH. We defined having ever been screened for VH as being previously tested, regardless of the VH type tested. We defined a participant as being “vaccinated” if he or she reported at least one (lifetime) previous dose of HBV vaccine and as “fully vaccinated” if he or she completed all three doses.

### Statistical analysis

All analyses were undertaken using the complex survey functions included in Stata 11 (StataCorp LP, College Station, TX, USA), which can incorporate weighting, clustering and stratification of data. Regarding the knowledge indicators, we reported proportions using 95 % confidence intervals (CIs) for male and female participants, who were further divided according to age group and socioeconomic status. We explored the prevalence rates of the knowledge indicators, screening and hepatitis B vaccination practices within the PCAP according to age group and socioeconomic status, which were further stratified by sex.

To identify the factors associated with having ever reported VH, weighted bivariable and multivariable logistic regression models were used. Variables with unadjusted odds ratios (ORs) of < 0.25 were included in the initial multivariable model. A backward fitting procedure was used, and variables were retained in the model when the *P*-value of the likelihood ratio test was less than 0.05. The magnitudes of the associations were estimated with weighted OR and 95 % CIs.

### Ethics

The PCAP study was granted ethical approval from the National Research Ethics Committee (reference: 64485/12). The interviewers explained the survey contents to the participants, and each of the participants was asked to sign a consent form.

## Results

In total, 6000 men and 6000 women were interviewed. The estimated response rate for the PCAP 2013 was 91.7 %. In each geographic region, the age and sex distributions of the sample were compared with the 2010 Demographic Census population distribution, and small differences (less than 1 %) were identified.

### Knowledge of VH transmission

Table 1 illustrates the weighted age-specific knowledge of the enrolled men and women. Regarding knowledge indicators related to transmission, the proportion of individuals who correctly identified all transmission routes of both HBV and HCV was much lower than those who correctly identified the transmission routes of each individual infection. A higher proportion of women than men correctly identified that HBV and HBV/HCV can be transmitted through dental treatments, dialysis and endoscopy (29.9 % vs. 25.5 % for women, respectively, *p* = 0.007; 11.6 % vs. 9.5 % for men, respectively, *p* = 0.01). Among the women, a higher proportion of those aged between 25 and 49 years correctly identified that HCV can be transmitted through unprotected sexual intercourse, sharing needles and tattooing or piercing.

**Table 1:** Correct knowledge related to viral hepatitis transmission among individuals aged between 15 and 64 years according to sex and age in Brazil as measured by the PCAP-2013

		Men					Women				
	Total	15–24 years	25–49 years	50–64 years	All age groups	*p*-value	15–24 years	25–49 years	50–64 years	All age groups	*p*-value
Unprotected sexual intercourse											
HBV	33.1 (30.3–35.9)	41.6 (33.8–49.9)	37.3 (32.5–42.4)	32.7 (26.0–40.2)	37.5 (33.0–42.3)	0.17	27.9 (22.1–34.5)	30.1 (26.0–34.7)	23.9 (18.6–30.1)	28.6 (25.4–32.0)	0.27
HCV	34.3 (31.6–37.0)	33.1 (26.4–40.6)	36.8 (32.2–41.8)	30.6 (24.1–37.9)	34.9 (31.1–38.9)	0.29	27.5 (21.5–34.4)	38.2 (33.5–43.2)	25.6 (19.9–32.1)	33.7 (30.3–37.3)	0.002
HBV/HCV	17.1 (14.8–19.5)	17.2 (12.1–23.7)	19.3 (15.6–23.7)	18 (13.0–24.3)	18.6 (15.3–22.4)	0.72	12.8 (8.7–18.4)	17.7 (14.3–21.8)	11.6 (7.9–16.6)	15.6 (13.0–18.6)	0.06
Sharing needles											
HBV	30.0 (27.5–32.6)	25.8 (20.4–32.1)	32 (27.7–36.8)	29.7 (23.5–36.7)	30.1 (65.8–34.2)	0.13	29.4 (23.9–35.6)	29.9 (26.2–33.9)	30.8 (24.7–37.7)	29.9 (26.8–33.2)	0.95
HCV	27.0 (24.7–29.3)	25.8 (20.3–32.2)	28.5 (24.4–32.9)	25.2 (19.7–31.7)	27.3 (23.9–30.8)	0.57	21.5 (16.8–27.2)	30.1 (26.5–34.0)	21.0 (16.3–26.6)	26.6 (23.9–29.5)	0.004
HBV/HCV	12.0 (10.3–13.8)	10.4 (6.8–15.4)	13.5 (10.9–16.7)	12.6 (8.4–18.6)	12.6 (10.3–15.4)	0.43	10.3 (6.9–15.2)	12.3 (9.9–15.1)	9.6 (6.6–13.9)	11.4 (9.5–13.7)	0.45
Sharing manicure/pedicure equipment											
HBV	30.4 (28.2–32.6)	30 (23.8–37.0)	30 (25.8–34.5)	31.4 (25.6–37.9)	30.2 (26.6–34.1)	0.90	31.5 (27.1–36.2)	31.7 (28.5–35.0)	25.2 (20.9–30.0)	30.6 (28.0–33.3)	0.06
HCV	27.2 (25.0–29.4)	31.1 (24.9–38.0)	28.8 (25.1–32.8)	23.4 (18.5–29.0)	28.4 (25.2–31.8)	0.15	24.2 (20.0–28.9)	28.0 (24.8–31.4)	23.2 (19.2–27.8)	26.3 (23.8–29.0)	0.12
HBV/HCV	9.7 (8.2–11.2)	11.3 (7.1–17.6)	10.5 (8.1–13.6)	11.8 (8.4–16.3)	10.9 (8.7–13.6)	0.85	7.7 (5.5–10.9)	9.4 (7.4–11.7)	8.2 (5.9–11.2)	8.8 (7.3–10.6)	0.52
Dental treatment, dialysis or endoscopy											
HBV	28.0 (25.6–30.4)	24.7 (19.0–31.5)	25.4 (21.7–29.5)	28.9 (22.7–35.9)	25.9 (22.6–29.5)	0.56	29.0 (23.3–35.4)	30.1 (26.4–34.2)	30.2 (24.4–36.6)	29.9 (26.7–33.3)	0.93
HCV	27.4 (25.1–30.0)	27 (21.0–33.9)	30.3 (26.2–34.8)	22.7 (17.6–28.8)	28.2 (24.8–31.2)	0.10	24.6 (19.5–30.6)	27.9 (24.1–32.1)	24.5 (19.5–30.3)	26.6 (23.6–29.9)	0.42
HBV/HCV	10.6 (9.0–12.2)	8.6 (5.3–13.6)	9.1 (7.0–11.9)	11.6 (7.8–16.9)	9.5 (7.6–11.8)	0.53	9.5 (6.4–13.9)	12.5 (9.9–15.7)	10.9 (7.6–15.5)	11.6 (9.5–14.1)	0.37
Tattooing or piercing											
HBV	26.4 (24.0–28.7)	22.5 (17.3–28.8)	26.4 (22.5–30.8)	27.0 (21.0–34.1)	25.6 (22.3–29.2)	0.43	23.5 (18.6–29.2)	29.3 (25.6–33.4)	23.6 (18.7–29.5)	27.1 (24.1–30.3)	0.07
HCV	24.5 (27.1–31.8)	30.3 (23.8–37.7)	28.9 (24.8–33.3)	28.3 (22.1–35.5)	29.1 (25.7–32.9)	0.89	23.6 (18.6–29.4)	33.4 (29.6–37.4)	25.1 (20.0–31.1)	29.8 (26.932.8)	0.003
HBV/HCV	11.3 (9.6–13.0)	7.9 (4.9–12.5)	10.3 (7.8–13.5)	11.8 (7.9–17.0)	10.0 (7.9–12.5)	0.37	9.0 (6.0–13.4)	15.1 (12.3–18.4)	7.9 (5.3–11.6)	12.5 (10.4–15.0)	0.001
Denominators											
Unweighted	12000	1503	3002	1495	6000		1499	3002	1499	6000	
Weighted	12000	1577	3208	1081	5867		1565	3362	1207	6133	

Percentages indicate those with correct knowledge related to viral hepatitis transmission. The data in parentheses are 95 % CIs

**Fig. 1:**
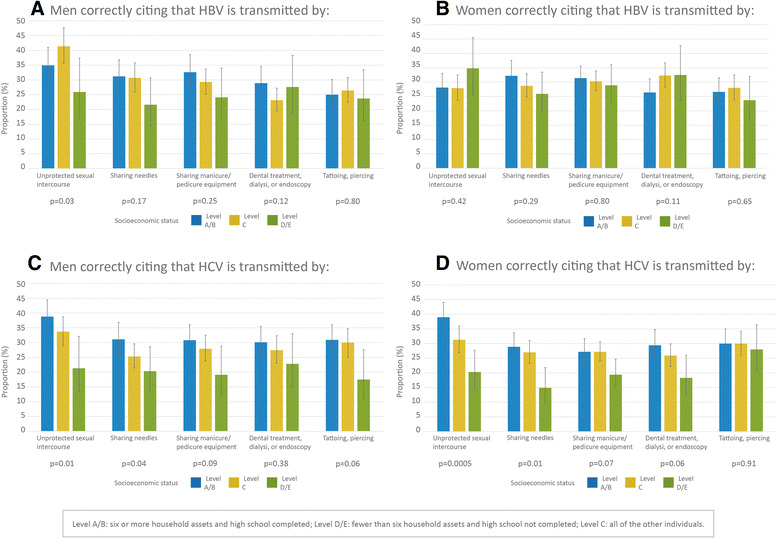
Knowledge related to viral hepatitis transmission among individuals aged between 15 and 64 years according to socioeconomic status and social class in Brazil as measured by the PCAP-2013

Among all of the socioeconomic status groups, the men with C-level socioeconomic status exhibited the highest level of knowledge that unprotected sex is a transmission route for HBV. For HCV, the proportions of men and women who correctly reported that unprotected sex and sharing needles are transmission routes for infection decreased as socioeconomic status decreased (Fig. 1).

### Screening for VH and vaccination against HBV

Overall, 29.0 % of the individuals (23.7 % of the men and 34.0 % of the women) reported having been screened for VH (Table 2). Among these respondents, 44.6 % were screened for HBV, 32.0 % were screened for HCV, and 16.8 % were screened for HDV. The proportion of participants who reported VH screening within the previous 12 months was higher among the men than the women (39.1 % vs. 34.3 %, respectively, *p* = 0.004). Additionally, among the women, this proportion decreased significantly with age. Overall, 17.5 % of the participants had been screened with a rapid test. The proportion of women who reported having been screened for the virus using rapid testing significantly decreased with age. Overall, 89.9 % of the participants who had been screened were aware of the results of their last VH test and 38.7 % knew of a service that provided testing for HBV and HCV free of charge. This proportion was higher among the women than the men (45.1 % vs. 32.1 %, respectively) and varied with age in both genders. The majority of the respondents (73.9 %) declared that they had been vaccinated against HBV (irrespective of the number of vaccination doses administered), and 18.3 % declared that they had completed the three-dose regimen.

**Table 2:** Screening for viral hepatitis and hepatitis B vaccinations among individuals aged between 15 and 64 years according to sex and age in Brazil as measured by the in the PCAP-2013

		Men					Women				
	Total	15–24	25–49	50–64	All age groups	*p*-value	15–24	25–49	50–64	All age groups	*p*-value
VH screening, ever	29 (27.6–30.4)	14.3 (11.9–17.1)	27.7 (25.6–29.9)	25.5 (22.3–28.9)	23.7 (22.1–25.3)	<0.001	24.5 (21.6–27.6)	41 (38.4–43.6)	26.6 (23.8–29.6)	34 (32–36)	<0.001
HBV	44.6 (42.2–47.2)	46.2 (37.2–55.5)	47.4 (42.9–52)	39.9 (33–47.3)	45.8 (42–49.6)	0.25	49.4 (42.9–55.9)	43.6 (39.8–47.5)	38.5 (32.5–44.8)	43.9 (40.1–47.2)	0.05
HCV	32 (29.7–34.5)	36.3 (27.6–46.1)	33.5 (29.2–38)	32.8 (26.4–40)	33.8 (30.3–37.6)	0.79	29.3 (23.2–36.4)	31.5 (27.9–35.4)	29.8 (24.1–36.2)	30.8 (27.7–34.1)	0.77
HDV	16.8 (13.7–19)	15.7 (10–23.8)	16.8 (13.8–20.3)	14.5 (10.3–20.1)	16.2 (16.7–19)	0.77	17.6 (12.4–24.3)	16.9 (14.1–20.1)	14.9 (10.8–20.2)	16.7 (14.2–19.6)	0.73
VH screening, last 12 months	36.2 (35.5–42.7)	38.5 (29.7–48)	40 (35.7–44.5)	36.3 (29.5–43.8)	39.1 (35.5–42.7)	0.70	45 (38.4–51.7)	33.2 (29.5–37.1)	26.3 (21–32.3)	34.3 (31.4–37.2)	0.0003
VH screening using rapid testing, ever	17.5 (15.6–19.6)	16.2 (10.1–25.1)	21 (17.3–25.2)	16.3 (11.8–22.2)	19.3 (16.5–22.4)	0.31	24 (18.2–31.1)	14.7 (12.1–17.8)	14.1 (10.1–19.3)	16.3 (13.8–19.2)	0.002
Knowledge of last VH test results	89.9 (88.3–91.3)	84.2 (75.2–90.4)	88.7 (84.7–91.8)	89.9 (84.3–93.7)	88.3 (85.2–90.7)	0.36	89.8 (85.5–93)	91.4 (89–93.3)	90.2 (86.6–93)	90.9 (89–92.5)	0.61
Knowledge of any health service that offers free screening for VH	38.7 (37–40.5)	26 (23–29.2)	34.6 (32.1–37.3)	33.3 (29.9–36.9)	32.1 (30–34.2)	<0.001	36 (32.8–40)	50.4 (47.6–53.3)	42.3 (38.9–46)	45.1 (42.7–47.5)	<0.001
HBV vaccination (1 dose)	73.9 (72.4–75.4)	68.8 (65.3–72)	71.3 (68.6–73.8)	72.2 (68.8–75.4)	70.8 (68.5–73)	0.19	76.8 (73.9–79.5)	76.4 (74–78.6)	78.6 (75.8–81.2)	76.9 (75–78.8)	0.38
HBV vaccination (3 doses)	18.3 (17.0–19.6)	10.3 (8.4–12.5)	14.3 (12.6–16.3)	12.8 (10.6–15.3)	13.0 (11.6–14.5)	0.01	24.7 (22.0–27.6)	24.6 (22.2–27.2)	17.9 (15.5–20.5)	23.3 (21.3–25.5)	<0.001
Denominators											
Unweighted	12000	1503	3002	1495	6000	…	1499	3002	1499	6000	…
Weighted	12000	1577	3208	1081	5867	…	1565	3362	1207	6133	…

... not statistically significant

**Fig. 2:**
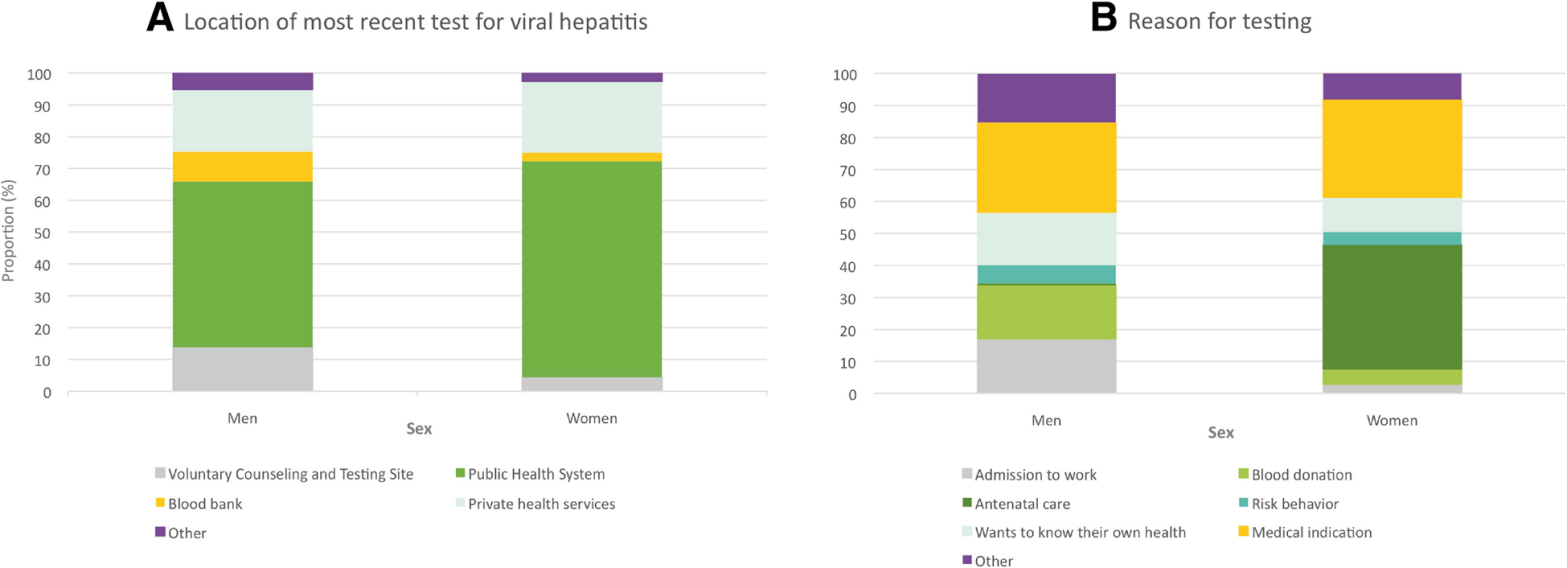
Location of most recent test for viral hepatitis and reasons for testing according to sex

Figure 2 illustrates the distribution of testing sites for the most recently recorded VH tests and details the reasons for testing according to sex. The majority of the respondents were tested in public hospitals or via primary health services (60.0 %), while 22.9 % were tested in private health services, and 6.2 % were tested at voluntary counselling and testing (VCT) sites. When dividing the respondents by sex, greater proportions of men reported having been last tested at a VCT site (13.8 % vs. 4.4 %) or a blood bank (9.4 % vs. 2.7 %). The majority of patients were screened because of a medical indication (29.7 %). Regarding the reasons for screening, greater proportions of men than women reported work-related reasons (17.1 % vs. 2.9 %, respectively) and blood donation (4.7 % vs. 1.6 %, respectively).

Table 3 illustrates the results for the variables associated with having been screened for VH. The proportion of men who reported undergoing screening for VH was greatest among those aged between 50 and 64 years; among the women, this proportion was greatest among those aged between 25 and 49 years. Among the men, screening increased significantly with age, whereas among the women the adjusted OR was highest for the 25- to 49-year-old group (AOR: 1.36, [95 % confidence interval: 1.10–1.67]) and decreased thereafter. Among the men, initial sexual intercourse before 15 years of age was significantly associated with VH screening. For both the men and the women, reported screenings decreased significantly with decreasing socioeconomic status. Overall, the proportions of men and women who reported undergoing VH screening were higher among those who had been tested for HIV, and this association remained after adjustment of variables.

**Table 3:** Reporting of hepatitis screening in relation to demographic status, behavioural variables and HIV testing history according to sex

	Men			Women		
	Percentage reporting hepatitis screening	Crude OR	Adjusted OR	Percentage reporting hepatitis screening	Crude OR	Adjusted OR
All	23.7 (22.1–25.3)			34 (32–36)		
Demographic characteristics						
Age groups						
15 − 24 years	14.3 (11.9 − 17.1)	1	1	24.5 (21.6 − 27.6)	1	1
25 − 49 years	27.7 (25.6 − 29.9)	2.29 (1.83 − 2.87)	1.33 (1.03 − 1.71)	41 (38.4 − 43.6)	2.14 (1.79 − 2.56)	1.36 (1.10 − 1.67)
50 − 64 years	25.5 (22.3 − 28.9)	2.05 (1.59 − 2.64)	1.52 (1.13 − 2.03)	26.6 (23.8 − 29.6)	1.12 (0.90 − 1.38)	1.29 (1.02 − 1.63)
Socioeconomic status						
A/B	30.6 (27.4 − 34)	2.38 (1.81 − 3.13)	1.78 (1.34 − 2.37)	40.6 (36.9 − 44.5)	2.35 (1.89 − 2.94)	2.10 (1.66 − 2.68)
C	22 (20 − 24.1)	1.52 (1.18 − 1.94)	1.14 (0.88 − 1.47)	34.4 (32 − 36.9)	1.80 (1.49 − 2.18)	1.56 (1.27 − 1.93)
D/E	15.7 (12.9 − 18.9)	1	1	22.5 (19.8 − 25.5)	1	1
Sexual behaviour						
Age at initial intercourse						
< 15 years	28.1 (25.4 − 30.9)	1.32 (1.23 − 1.60)	1.32 (1.10 − 1.57)	33 (29.2 − 37.1)	0.83 (0.68 − 1.00)	
≥ 15 years	22.8 (20.9 − 24.8)	1	1	37.4 (35.1 − 39.7)	1	…
Sex with same sex, ever						
No	24.4 (22.6 − 26.1)	1		36.6 (34.5 − 38.9)	1	
Yes	30 (24.5 − 36.1)	1.33 (1.00 − 1.77)		38.6 (31.1 − 46.6)	1.08 (0.78 − 1.51)	…
Condom use at last sexual intercourse						
Yes	24.6 (22.2 − 27.3)	1		38.2 (34.7 − 41.9)	1	
No	26.7 (24.3 − 29.2)	1.11 (0.93 − 1.35)	…	39.1 (36.4 − 41.7)	1.03 (0.87 − 1.23)	…
Number sexual partners over the lifetime						
≤ 10	21.7 (19.2 − 24.3)	1		42.1 (39.2 − 45)	1	
> 10	27.9 (25.4 − 30.6)	1.40 (1.16 − 1.69)	…	38.7 (33.8 − 43.9)	0.87 (0.70 − 1.09)	…
Having casual partner, last 12 months						
No	27 (24.9 − 29.2)	1		39.7 (37.3 − 42.1)	1	
Yes	23.6 (21 − 26.4)	0.84 (0.70 − 0.99)	…	33.6 (28.8 − 38.7)	0.76 (0.61 − 0.96)	…
Intravenous drugs, ever						
No	23.8 (22.2 − 25.5)	1		34 (32 − 36)	1	
Yes	19.9 (10.1 − 35.4)	0.80 (0.36 − 1.76)	…	29.7 (18 − 44.8)	0.82 (0.43 − 1.58)	…
Tested for HIV, ever						
No/don’t know	13 (11.6 − 15.6)	1	1	16.5 (14.8 − 18.4)	1	1
Yes	53.8 (50.4 − 57.1)	7.79 (6.46 − 9.39)	7.05 (5.82 − 8.53)	60.1 (57.1 − 63)	7.62 (6.44 − 9.00)	7.13 (5.97 − 8.54)

The data in parentheses are 95 % CIs

*COR* crude odds ratio, *AOR* adjusted odds ratio

... not statistically significant

## Discussion

This study is the first to report the low level of knowledge related to HBV and HCV transmission in Brazil and the low screening rates for VH within the general population. The strengths of this study are the high response rate and large probability sample, which reflected a broad Brazilian population in terms of demographic characteristics.

For all of the assessed knowledge indicators, fewer than 35 % of the participants correctly identified the transmission routes for both HBV and HCV. Although the major HBV transmission route in Brazil is sexual intercourse [13], only 33.1 % of the respondents correctly identified this transmission route. This percentage is lower than the percentages observed in other population-based studies; for example, 44 % of a sample from Hong Kong correctly identified this transmission route [20], while approximately 60 % of a sample in Germany [21, 22], 70 % of a sample in France [23], and 77.6 % of a sample in the Netherlands [21] provided correct identification.

In Brazil, donated blood is routinely screened for HCV, and measures are in place to facilitate infection control and safe-injection practices; thus, HCV is now transmitted primarily through injected drug use. The prevalence of markers of hepatitis B and C among intravenous drug users are high at 63.9 and 55.8 %, respectively [24]; however, knowledge related to the transmission of HCV through shared needles was low. A similar result was observed for HBV. These results are inconsistent with those reported by other studies. For example, 44 % of respondents in Hong Kong [20] and 95 % in Germany [22] correctly identified that HBV can be transmitted by sharing needles. A similar result was obtained for knowledge related to HBV transmission through tattooing and piercing, with much lower levels of knowledge reported in Brazil than in Hong Kong (37 %) [20] and Germany (73 %) [22].

Furthermore, the proportion of respondents who correctly identified the transmission routes of both HBV and HCV substantially dropped relative to the proportions of individuals who identified the transmission routes of the individual virus types. This confusion within the general population regarding the specificities associated with each hepatitis type has also been observed in previous studies [4, 20–22].

The commemoration of World Hepatitis Day (named in the 2010 resolution WHA63.18) [25] is helping to raise awareness and mobilize governments and resources to aid in the fight against VH in Latin America [14]. In Brazil, although World Hepatitis Day campaigns have been conducted since 2010, knowledge related to VH remains low. Because increasing the knowledge of HBV and HCV is an effective means of preventing or reducing the spread of infection [26–28], additional efforts are needed to improve public education regarding the transmission routes and specificities of HBV and HCV.

Our results revealed that there is an urgent need to improve overall testing uptake for VH. Such screening is a necessary first step toward providing access to prevention, care and treatment services. Only one third of the participants in this study had ever been screened for VH; this proportion was higher for HBV (44.6 %) than for HCV (32 %). Early diagnosis of VH is among the main priorities of the Department of STIs, AIDS and VH of the Brazilian Ministry of Health [29]. Since 2004, free testing for VH has been offered at VCT sites located in clinics and specialized centres [30], although only a small proportion of our participants (6.2 %) reported having been screened for VH at such sites. Members of the general population may be reluctant to undergo testing at these sites because the sites are designed to offer VH and HIV testing specifically for populations who are at a greater risk of exposure to HIV and other STIs, such as men who have sex with men, sex workers and intravenous drug users. Moreover, studies have shown that VCT sites exhibit low levels of performance with regards to installed capacity and present significant limitations in terms of the quality of diagnostic services and the promotion of prevention measures [31]. As a strategy to improve access to diagnosis, Brazil implemented rapid testing in 2011 within the public health system [30]. However, our results indicated that only 17.5 % of the respondents had been screened with such tests, which suggests that rapid testing has not been widely implemented. Only a small proportion of participants reported engagement in high-risk behaviours as a reason for testing, suggesting that testing was performed for reasons other than individual perceptions of risk, which is a factor that has been associated with not being tested in other studies.

Among the enrolled women, VH screening coverage was also low, which is surprising because such screening is offered during pregnancy: 96 % of pregnant women in Brazil receive antenatal care [32], and 83.5 % of pregnant women are tested for HIV [33]. Although this result is subject to respondent recall bias, this low reported coverage among women might be explained by a possible lack of information provided by health professionals to pregnant women with regard to VH screening. Providers might not always communicate VH screening results to women if they do not pose a threat to the patient or baby, such as when results are negative or indicate past infection. Another possible explanation is that many women might not be aware that they are being screened for VH during pregnancy. The provision of appropriate pre- and post-test counselling for VH during antenatal care should be improved by providing appropriate training to doctors and mid-wives [14].

In our multivariate analysis, the participants who reported having undergone VH screening were seven times more likely to have been tested for HIV compared with those who had never been tested for VH. One potential explanation for this finding is that the same services offer testing for both infections, which are a part of the screening package used to evaluate pregnant women and high-risk groups, such as sex workers and men who have sex with men [29]. The HIV testing rate (54 % in men and 60 % in women) was higher than the VH testing rate; however, despite this difference, the HIV testing rate was strikingly low considering that HIV prevention has been the subject of broad testing provisions, effective testing promotion at all levels of health services, testing campaigns and widespread community advocacy and awareness campaigns [34]. This result indicates that difficulties remain in overcoming the general population’s reluctance to seek testing, although it can be used as a lesson for VH advocacy.

The enrolled men who were aged between 50 and 64 years old were significantly more likely to have been screened. Several studies have identified an association between increased age [35, 36] and testing status, whereas other studies have not found this association [28]. Socioeconomic differences were observed in various aspects of this study. Multivariable analysis identified associations between lower socioeconomic status and a lack of screening in both men and women. Moreover, the participants of lower socioeconomic status exhibited generally poorer knowledge of VH transmission, a finding that has been corroborated by several authors [20, 21]. This lack of knowledge is particularly important because the prevalence of VH is higher in individuals in economically and socially vulnerable situations [3, 23].

We found that 73.9 % of the respondents had received hepatitis B vaccines. This proportion is higher than those reported in previous population-based studies conducted in other countries [20, 22, 23]. However, the proportion of respondents who had completed all three doses of the vaccine was considerably lower at 18.3 %. In this study, no vaccination cards were verified or cross-referenced and therefore this result is subject to respondent recall bias. Importantly, 30 % of the men and 23 % of the women under 24 years of age had not been vaccinated against HBV, and sexual transmission of HBV remains a concern among unvaccinated adolescents in Brazil. Nevertheless, vaccination against HBV was extended to young individuals (aged 20–24 years) in 2011 to increase the coverage rate.

The current study has several limitations. The PCAP-2013 relied on self-reported data, which are subject to recall and desirability biases. These biases might have influenced the high rate of HBV vaccine coverage, which was not cross-checked against vaccination records. Additionally, questions related to certain behaviours were sensitive and might have been under-reported. We sought to minimize this bias by using a self-interview technique for the survey sections regarding sexual practices and drug use. Several studies have demonstrated that it is difficult for the general population to differentiate between various types of VH [4, 20, 22]. Consequently, interpretations of specific knowledge indicator values should be assessed with caution [20]. However, our findings are valuable as baseline information and can be assessed in future rounds of the PCAP, which should take into account changes in census tracts when establishing a longitudinal comparison.

## Conclusions

The PCAP-2013 provides a novel assessment of VH in Brazil. In measuring the extent of knowledge related to VH across a large representative sample, we found that our study participants possessed low levels of knowledge related to hepatitis B and C. This study also underscores the need to improve screening practices used in the general population and to specifically target younger and lower socioeconomic status populations. Based on these findings, current preventive measures must be reinforced. Efforts to enhance education through awareness campaigns, increased testing resources and expanded testing sites are needed to remove barriers to testing and improve access to treatment.

## Abbreviations

ABEP: Association of Research Companies
AOR: adjusted odds ratio
CIs: confidence intervals
FMT-HVD: Fundação de Medicina Tropical Doutor Heitor Vieira Dourado
HAV: hepatitis A virus
HBV: viral hepatitis B
HCV: hepatitis C virus
HDV: hepatitis D virus
HEV: hepatitis E virus
OR: odds ratio
PCAP: Survey of Knowledge, Attitudes and Practices of HIV and Other Sexually Transmitted Infections
PCAP-2013: Third round of the Survey of Knowledge, Attitudes and Practices of HIV and Other Sexually Transmitted Infections
PSUs: primary sampling units
STIs: sexually transmitted infections
VCT: voluntary counselling and testing
VH: viral hepatitis
